# Exploring Multiple Aspects of Taxonomic and Functional Diversity in Microphytobenthic Communities: Effects of Environmental Gradients and Temporal Changes

**DOI:** 10.3389/fmicb.2021.668993

**Published:** 2021-05-21

**Authors:** Anette Teittinen, Leena Virta

**Affiliations:** ^1^Department of Geosciences and Geography, University of Helsinki, Helsinki, Finland; ^2^Tvärminne Zoological Station, University of Helsinki, Hanko, Finland

**Keywords:** alpha diversity, beta diversity, diatoms, environmental gradients, functional diversity, rock pools

## Abstract

Biodiversity has traditionally been quantified using taxonomic information but the importance of also considering its functional characteristics has recently gained an increasing attention among microorganisms. However, studies exploring multiple aspects of taxonomic and functional diversity and their temporal variations are scarce for diatoms, which is one of the most important microbial groups in aquatic ecosystems. Here, our aim was to examine the taxonomic and functional alpha and beta diversities of diatoms in a coastal rock pool system characterized by a naturally high environmental heterogeneity. We also investigated the temporal differences in the diversity patterns and drivers. The relationship between the species richness and functional dispersion was temporally coherent, such that species-poor communities tended to be functionally clustered. The trend between the species richness and taxonomic uniqueness of community composition was temporally inconsistent, changing from negative to non-significant over time. Conductivity or distance to the sea or both were key determinants of species richness, functional dispersion, and uniqueness of community composition. The increase of community dissimilarity with an increasing environmental distance was stronger for the taxonomic than the functional composition. Our results suggest that even minor decreases in the species richness may result in a lowered functional diversity and decreased ecosystem functioning. Species-poor ecosystems may, however, have unique species compositions and high contributions to regional biodiversity. Despite changing the species compositions along the environmental gradients, communities may remain to have a high functional similarity and robustness in the face of environmental changes. Our results highlight the advantage of considering multiple biodiversity metrics and incorporating a temporal component for a deeper understanding of the effects of environmental changes on microbial biodiversity.

## Introduction

During the era of ongoing global change, the need to understand biodiversity patterns has increased (McGill et al., 2015). Biodiversity is a broad concept, which can be measured by multiple metrics at different spatial scales, such as alpha diversity, that is, species richness, describing the number of species in a local community; and beta diversity (Whittaker, 1960), which can be defined as the differences in community composition between local communities. Although the trends in species richness are widely used to assess the responses of biological communities to environmental change, they are often not connected to the rates of change in community composition, and may, thus, fail to capture the key elements of biodiversity change in changing environments (Hillebrand et al., 2018). Therefore, beta diversity should also be considered to understand the mechanisms influencing regional biodiversity and for guiding efforts to conserve it (Socolar et al., 2016).

Traditionally, measures of taxonomic diversity have served as a basis for quantifying biodiversity. However, considering only the taxonomic characteristics of communities ignores the different functions that species have in an ecosystem. Thus, the inclusion of the functional characteristics (i.e., traits) can provide a more effective way to describe diversity (Mouchet et al., 2010). Functional traits describe the performance of an organism, that is, how the organism acquires resources and tolerates stressors (Violle et al., 2007) and, hence, traits provide a link between the community and environment. Studying functional diversity also allows comparisons among different ecosystems, such as rock pools and other aquatic ecosystems, to be made despite the differences in the taxonomic compositions. For a deeper understanding of the patterns and processes influencing communities, using trait-based approaches in combination with taxonomic information may be particularly useful (Leibold and Chase, 2018).

The importance of exploring not only the taxonomic, but also the functional aspects of biodiversity has been increasingly recognized in the research of microbial organisms. In many aquatic ecosystems, one of the most important microbial groups are the diatoms, which is a diverse group of unicellular algae that are important contributors to primary production and have essential roles in food webs. In most aquatic environments, diatoms show a high taxonomical diversity comprising many species with specific environmental preferences; therefore, they are widely used to assess environmental conditions (Smol and Stoermer, 2010). Functionally, different diatom species can be distinguished by their growth habits, which have developed as responses to, for instance, certain habitats, attachment, or nutrient capture (Round et al., 1990; Rimet and Bouchez, 2012). For example, species may have adapted to live in the benthic environment, in which they may be mobile or live attached to the substrata by various ways. Depending on their tolerance toward physical disturbances and ability to utilize nutrient resources, species with different growth habits can be further assigned into ecological guilds (Passy, 2007). For instance, low-profile species are considered tolerant to physical disturbances and adaptive to low-nutrient conditions; whereas high-profile species are favored in high-nutrient conditions, yet are susceptible to disturbances. The guild classification has been widely used in the ecological studies of benthic diatoms (reviewed in Tapolczai et al., 2016).

Recently, diatom traits have been successfully used as indicators of changing ecological conditions in benthic systems in terms of, for instance, organic pollution and trophic levels (Berthon et al., 2011), nutrients and light (Lange et al., 2011), and hydrological disturbances (Wu et al., 2019). The usefulness of a trait-based approach to reveal the environmental changes was also highlighted in a global-scale study, in which, the diatom functional composition responded more strongly to the environmental gradients than the taxonomic composition (Soininen et al., 2016). Despite an increased interest in the functional approaches among diatoms, studies exploring both the alpha and beta diversities with the taxonomic and functional data are scarce. For rock pool systems, such studies for diatoms are entirely lacking and, also, microphytobenthos, in general, has received little attention in these ecosystems. Rock pools are water-filled depressions in a bedrock occurring in areas where the bedrock surface is exposed owing to, for instance, wind, and wave actions (Pajunen and Pajunen, 2007). In many regions along the coast of the Baltic Sea, rock pools provide widespread patchy aquatic habitats. By serving suitable habitats for the aquatic biota within a terrestrial landscape, they provide essential ecosystem functions (De Meester et al., 2005) and contribute to the maintenance of the aquatic biodiversity. Rock pools are typically small and structurally simple systems, yet they exhibit a high environmental variability over relatively small spatial extents (Pajunen and Pajunen, 2007). They provide excellent model systems for studying general ecological questions (Ebert et al., 2001; Langenheder et al., 2012), and incorporating the functional traits can provide complementary information into their roles as such.

Here, we present a study on the taxonomic and functional alpha and beta diversities of benthic diatom communities along steep environmental gradients in coastal rock pool ecosystems. Specifically, we ask whether there is a relationship between local species richness and functional diversity. Rapid loss of functional diversity as species diversity declines would suggest a low functional redundancy, whereas high levels of redundancy would imply ecosystem functions that are robust to diversity changes (Micheli and Halpern, 2005). We also investigate whether there is a relationship between species richness and taxonomic uniqueness of community composition, testing the idea that communities with certain species diversity levels would contribute more strongly than others to the overall taxonomic beta diversity. A negative relationship between the metrics would indicate that species-poor communities often have a high uniqueness of community composition (Legendre and De Cáceres, 2013), and hence, preserving systems with a low number of species may be essential for maintaining regional biodiversity. Furthermore, we compare the levels of taxonomic and functional beta diversity and examine whether there are differences in the change of taxonomic and functional community dissimilarities with increasing environmental distances. We also ask what are the key factors shaping the taxonomic and functional diversity, and whether the observed patterns and key determinants exhibit differences between the two time periods. Compared to snapshot studies, incorporating a temporal aspect provides a more thorough understanding about the mechanisms influencing the communities (Langenheder et al., 2012), and facilitates the identification of generalities in biodiversity patterns over time. Although a few studies have addressed the temporal variation in rock pool microbial communities using taxonomic information (e.g., Langenheder et al., 2012; Aarnio et al., 2019), to our knowledge, no study has assessed such trends based on the functional data.

## Materials and Methods

### Study Area and Rock Pools

The study region is located in southern Finland, on the coast of the Baltic Sea (59°48′ to 59°51′N, 23°12′ to 23°18′E; Supplementary Figure 1). Sampling took place on ten islands, and comprised of 91 rock pools in total. Within each island, the aim was to sample a wide range of different pool types, including freshwater pools that are located farther away from the seashore and more exposed brackish pools at a closer proximity to the sea, to capture as high environmental heterogeneity as possible. We sampled each pool once in June and once in September–October (from now on September) 2018. In both occasions, sampling was carried out within ca. 2 weeks. Between the sampling periods, the prevailing weather conditions differed. The average of the daily mean air temperatures was higher in June, whereas the precipitation sum and average of daily mean wind speed were higher in September (Supplementary Table 1; Finnish Meteorological Institute, 2020). During the sampling, the pools were unshaded and not connected to each other or the sea by permanent watercourses. In September, a few pools close to the shore were subject to the occasional inputs of seawater.

### Field Sampling and Laboratory Methods

At each pool, we collected diatoms by scraping the submerged surface of the bedrock with a sponge (∼2 cm × 2 cm × 2 cm). A separate sponge was used at each rock pool. Ten subsamples of 100 cm^2^ were scraped from different sides of the pool and combined into a composite sample at each pool. The samples were preserved with ethanol and stored in a cold (+4°C) and dark environment until the laboratory analyses.

In the field, we measured the water pH, conductivity, and temperature using the Hach HQ40d multimeter and collected water samples, which were analyzed later in the laboratory for total nitrogen (TN) according to standard SFS-EN ISO 11905–1 and; for total phosphorus (TP), according to standard SFS-EN ISO 6878. We recorded the pool dimensions by measuring the maximal length, maximal width (perpendicular to length), and maximal depth; and subsequently calculated the pool surface area (length × width) and estimated pool volume as a shape of an inverted pyramid (length × width × depth/3; Ebert et al., 2001). We also measured each pool’s shortest distance to the seashore and recorded pool elevation with a GPS. Pool locations were acquired using a GPS, orthophotos, and digital maps. We also measured the shortest distance to the mainland of Finland from each pool using digital maps and orthophotos.

### Diatom Analysis

The diatom samples were cleaned of organic material in the laboratory using wet combustion with hydrogen peroxide (30% H_2_O_2_) and mounted on slides with Naphrax. Then, using a phase contrast light microscope (Olympus BX40, Melville, NY, United States) with a 1,000 × magnification, vertical non-overlapping transects on the slides were scanned until ∼500 frustules per sample were counted and identified to the lowest possible taxonomic level (mostly species level) following Krammer and Lange-Bertalot (1986, 1988, 1991a,b), Snoeijs (1993), Snoeijs and Vilbaste (1994), Snoeijs and Potapova (1995), Snoeijs and Kasperovicienè (1996), and Cantonati et al. (2017). Subsequently, the counts were transformed into species relative abundances (%), and species richness was computed as the sum of all taxa recorded in each pool. The estimation of species richness is therefore sample-based, and the true diatom species richness in each rock pool is perhaps higher because more species would likely be found by increasing the counting effort (Gotelli and Colwell, 2001). To assess the relationship between the number of species and counting effort, we conducted a rarefaction analysis. The results showed that while the subsamples of 500 frustules may somewhat underestimate the diatom species richness of the most diverse communities, they seem to represent the richness of the less diverse communities well (Supplementary Figure 2). We believe that our counting effort is thus sufficient for representing the true diatom community patterns here. Diatom taxonomic community composition in each rock pool in June is available in Supplementary Data 1, and for September, in Supplementary Data 2.

For the functional diversity analyses, the diatom taxa were grouped according to their life-forms, cell sizes, and ecological guilds. Based on the life-forms, taxa were classified according to the mobility (mobile or non-mobile), mode of attachment (adnate or pad-attached or stalk-attached or non-attached), and ability to form colonies (colonial or non-colonial); and based on the cell size, the diatoms were divided into small (biovolume < 1,000 μm^3^) and large (biovolume > 1,000 μm^3^) taxa (Snoeijs et al., 2002; Rimet and Bouchez, 2012). Based on the ecological guilds, they were classified into low-profile, high-profile, motile, and planktonic (Passy, 2007; Rimet and Bouchez, 2012). The planktonic guild comprises of taxa that are adapted to live in lentic environments (Rimet and Bouchez, 2012) but may inhabit benthic biofilms due to sedimentation (Soininen et al., 2016). Hence, this guild is also often included in studies which were focused on benthic diatoms (e.g., Soininen et al., 2016; Perez Rocha et al., 2019; Wu et al., 2019; Virta et al., 2020). Finally, the taxa were grouped according to their ability to fix nitrogen (nitrogen-fixing or non-nitrogen-fixing; Soininen et al., 2016). In addition to the cited references, Diatoms of North America (2019) was used to assign traits for some of the diatom taxa. Any given taxon may be assigned into several trait categories. Trait classifications for the diatom taxa observed in June are available in Supplementary Data 3; and for the diatom taxa observed in September, in Supplementary Data 4. For the analyses of the functional community composition, the functional composition data were constructed by summing the relative abundances of all the species in each of the trait categories. The traits used here include the species morphological characteristics, and are tightly connected to ecosystem functioning. For instance, grazers are dependent on species that have a high-profile growth form, whereas species with a low-profile growth form persist in low nutrients conditions, which are unfavorable to high-profile and motile species (Passy, 2007). Hence, species with a low-profile growth form may also support biomass production under such conditions. Despite the fact that the trait classifications used here include relatively few traits, to our knowledge, they comprise the most well-defined classifications for diatoms, and have been reported to be useful in ecological studies focused on benthic systems.

### Statistical Analyses

Statistical dependence between the explanatory variables was assessed using the Spearman’s rank (*r*_*s*_) correlation coefficients. Rock pool elevation and distance to the sea were highly correlated (June *r*_*s*_ = 0.71; September *r*_*s*_ = 0.78); thus, we excluded elevation because we had more precise field measurements for the distance to the sea. Pool volume was correlated with pool depth (June *r*_*s*_ = 0.81; September *r*_*s*_ = 0.78) and area (June *r*_*s*_ = 0.96; September *r*_*s*_ = 0.97). We decided to use the pool volume in the statistical analyses to describe the pool size. In June, TN was excluded as it was strongly correlated with TP (*r*_*s*_ = 0.80). Although the correlation between TN and TP was not significant in September (*r*_*s*_ = 0.55), we also opted to use TP rather than TN in the analyses of the September data in order to compare the results between the two time periods. All other pairs of explanatory variables had correlation coefficients *r*_*s*_ ≤ 0.7. We did not use the pool water temperature in the data analyses because the values were highly influenced by the prevailing weather conditions and sampling time.

To measure the functional diatom diversity, we computed the functional dispersion (FDis; Laliberté and Legendre, 2010) for each rock pool. Functional dispersion is a multidimensional distance-based functional diversity index, which measures the individual species’ weighted mean distance to the weighted centroid of all species, where weights correspond to the species’ relative abundances (Laliberté and Legendre, 2010). By construction, the functional dispersion index is not influenced by species richness. The functional dispersion was computed using the relative abundance and trait data and the function *dbFD* in the R package FD (Laliberté and Legendre, 2010; Laliberté et al., 2014).

To assess the taxonomic uniqueness of rock pool diatom communities, we calculated the local contributions to beta diversity (LCBD; Legendre and De Cáceres, 2013). The LCBD indicates the relative contribution of individual sampling sites to the beta diversity, with high values indicating sites that have unique community compositions in the data. The metric measures the uniqueness of each site in terms of community composition and allows one to identify sites with above or below average contributions to the beta diversity. The LCBD values were calculated using the Hellinger-transformed abundance data and the function *beta.div* in the R package adespatial (Dray et al., 2019).

We explored the relationships between species richness and functional dispersion or LCBD with linear regression. To identify a potential non-linear pattern between the metrics, we also used models that comprised of the quadratic terms of species richness.

To identify the key variables explaining the variation in species richness, functional dispersion, and LCBD among the pools, we used the generalized linear models (GLMs) with a Gaussian error distribution. For each response variable, we first constructed a full model that comprised of all the environmental variables (i.e., conductivity, pH, TP, and pool volume), and distances to the sea and to the mainland as spatial factors. Then, the best approximating model was selected using a backward stepwise model selection (function *stepAIC* in the R package MASS; Venables and Ripley, 2002). To complement the GLM analyses, we also carried out the principal component analyses (PCA) to illustrate the main patterns of variation within the data comprising the explanatory variables used in the GLMs. Prior to the PCAs, conductivity, TP, pool volume, and distance to the sea were log_10_-transformed, and all variables were standardized.

To investigate the increase of community dissimilarity with environmental distance, dissimilarity matrices were first constructed for the taxonomic and functional community composition by calculating the pairwise community dissimilarities using the Bray–Curtis index on the relative abundance data. The distance matrices for environmental data were created by calculating the pairwise Euclidean distances of the environmental variables (i.e., conductivity, pH, TP, and pool volume). Prior to the analyses, the variables, other than the pH, were log_10_-transformed, and subsequently, all variables were standardized (mean = 0, SD = 1). Then, Mantel tests were used to assess the relationships between community dissimilarity and environmental distance. To guard against the potential confounding effects of spatial factors, we also ran partial Mantel tests to separate the pure effects of environmental distance on community dissimilarity, while controlling for the effects of spatial distances, which were calculated as the pairwise Euclidean distances of the pool coordinates. We also assessed the relationships between community dissimilarity and spatial distance using Mantel tests and tested for the pure spatial effects, while controlling for the effects of environmental distances with partial Mantel tests. All analyses were conducted separately using the taxonomic and functional data and the two time periods.

Finally, to assess the effects of rare species on the Mantel test results, we carried out the Mantel tests for the taxonomic composition twice. Firstly, we ran the analyses with data that were comprised of all the species and, secondly, with data that included the species occurring in at least ten pools. The exclusion of the rare species present in less than ten pools restricted the analysis to 32% of all species in June and 29% of all species in September. Because the results were qualitatively similar regardless of the method used, we present here the results obtained using the entire data with all the species. In all the Mantel tests, Pearson’s correlation was used, and the significances of the relationships were tested using 9,999 permutations. Mantel tests were carried out using the R package vegan (Oksanen et al., 2019).

All statistical analyses were performed separately for the June and September data and conducted with the R version 3.6.1 (R Core Team, 2019).

## Results

The studied rock pools showed a high spatial and temporal variation in environmental characteristics, covering gradients from freshwater to brackish, acid to alkaline, and oligo-mesotrophic to hypereutrophic (Supplementary Datas 5, 6). In general, pool water conductivity was higher in June (range: 0.04–43.63 mS cm^–1^; mean: 7.49) than in September (range: 0.03–9.15 mS cm^–1^; mean: 5.76). Water pH ranged from 4.9 to 10.3 (median: 9.2) in June and from 4.5 to 10.2 (median: 8.7) in September. TP concentrations were higher in June (range: 0.03–6.81 mg L^–1^; mean: 0.50) than in September (range: 0.008–0.74 mg L^–1^; mean: 0.07).

In total, 183 diatom taxa were identified in June and 192 in September. Local species richness showed a high variability, ranging from 6 to 60 in June (mean: 25) and from 7 to 67 in September (mean: 23). None of the species occurred in all the pools in either June or September. Four species, *Navicula perminuta*, *Tabularia fasciculata*, *Diatoma moniliformis*, and *Nitzschia microcephala*, were widely distributed at both time periods, occurring at ≥80% of the pools. Regarding the traits, majority of them were present at most communities, that is, ≥85% of traits at ∼90 and 93% of the communities in June and September, respectively. Traits that were often lacking from the communities were nitrogen-fixing ability and planktonic guilds in June, and nitrogen-fixing ability in September. Most of the observed species were benthic, and members of the planktonic guild comprised only 2.7 and 1.6% of species in June and September, respectively. The planktonic species also typically had low relative abundances among the rock pools in which they occurred (mean relative abundance range in June: 0.2–0.4%; September 0.3–3.9%).

**FIGURE 1:**
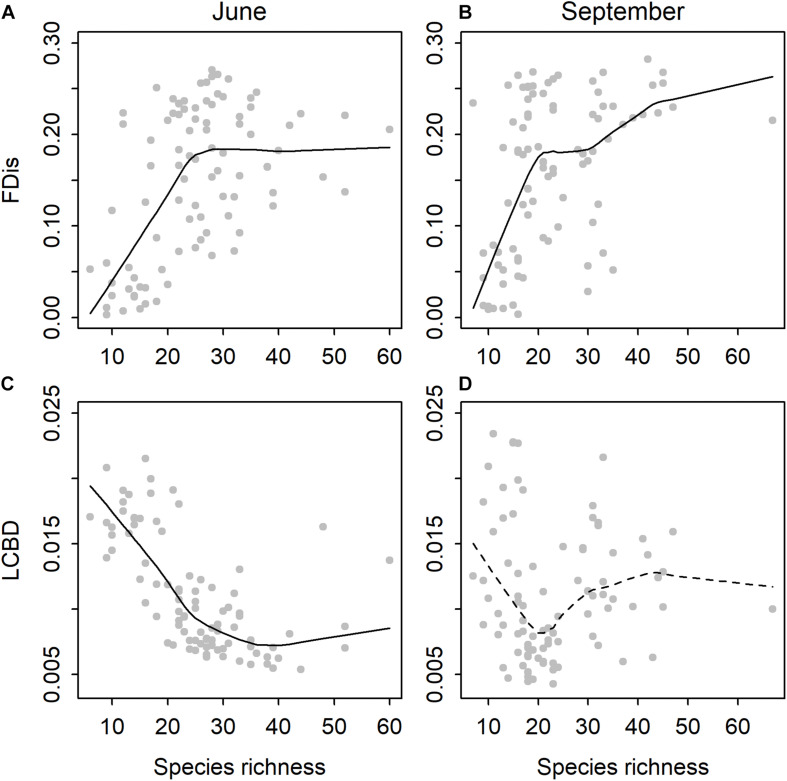
Relationships between species richness and functional dispersion (FDis) in **(A)** June and **(B)** September, and between species richness and local contributions to beta diversity (LCBD) in **(C)** June and **(D)** September for diatoms among the rock pools (*n* = 91).

There was a significant relationship between species richness and functional dispersion in both time periods (Figures 1A,B). The relationships were better explained with a non-linear (June: quadratic *R*^2^ = 0.33, *P* < 0.001; September: quadratic *R*^2^ = 0.22, *P* < 0.001) than a linear model (June: *R*^2^ = 0.23, *P* < 0.001; September: *R*^2^ = 0.19, *P* < 0.001). In June, there was a significant negative linear relationship between species richness and LCBD (*R*^2^ = 0.37, *P* < 0.001), although the trend was also slightly non-linear (quadratic *R*^2^ = 0.60, *P* < 0.001; Figure 1C). In September, species richness and LCBD were not related (linear *R*^2^ = 0, *P* = ns; quadratic *R*^2^ = 0.02, *P* = ns; Figure 1D).

According to the GLMs, species richness in June was most strongly associated with distance to the sea, which had a negative effect on species richness (Table 1). In September, the key variables affecting species richness were distance to the mainland and pH, both of which had negative influences. For LCBD, conductivity and distance to the sea were the most important variables in both time periods; LCBD decreased with increasing conductivity and increased with increasing distance to the sea. For functional diversity, the most important driving variable was conductivity, with a positive effect on functional dispersion in both June and September.

**TABLE 1:** Results of the generalized linear models to explain the variation in species richness, local contributions to beta diversity (LCBD), and functional dispersion of rock pool diatom communities (*n* = 91) in June and September.

	Variable	Estimate	SE	*t*-value	*P*-value	*D*^2^
**June**						
Species richness	Distance to sea	–0.6389	0.1727	–3.699	<0.001***	20.6
	Distance to mainland	–1.8855	1.1657	–1.618	0.109	
LCBD	Conductivity	–0.0002	0.0001	–3.011	0.003**	49.5
	pH	–0.0006	0.0003	–1.855	0.067	
	Distance to sea	0.0003	0.0001	4.760	<0.001***	
Functional dispersion	Conductivity	0.0043	0.0011	3.848	<0.001***	14.6
	pH	–0.0128	0.0078	–1.630	0.107	
**September**						
Species richness	Conductivity	0.7878	0.3257	2.419	0.018*	34.8
	pH	–6.1028	1.2610	–4.840	<0.001***	
	TP	0.0153	0.0091	1.677	0.097	
	Distance to mainland	–5.4009	1.1450	–4.717	<0.001***	
LCBD	Conductivity	–0.0008	0.0001	–5.809	<0.001***	54.7
	Distance to sea	0.0002	0.0001	3.005	0.003**	
	Distance to mainland	–0.0012	0.0005	–2.444	0.017*	
Functional dispersion	Conductivity	0.0091	0.0025	3.580	<0.001***	14.5
	pH	–0.0268	0.0104	–2.582	0.011*	

Estimates, standard errors (SE), *t*-values, and *P*-values showing variable significance (*P < 0.05, **P < 0.01, and ***P < 0.001) are given. *D*^2^ = Explained deviance of the model (%).

The PCA results showed that in June, the most important variable along the first axis was distance to the sea and conductivity along the second axis (Supplementary Figure 3). The first two axes explained 62.1% of the variation in June. In September, the most important factors on axis 1 were distance to the mainland and TP and conductivity on axis 2. The first two axes explained 62.2% of the variation in September.

Mean pairwise dissimilarities were higher for the taxonomic than the functional community composition. For the taxonomic composition, mean dissimilarity was 0.79 (range: 0.02–1.0) in June and 0.76 (range: 0.03–1.0) in September. For the functional composition, mean dissimilarity was 0.23 (range: 0–0.59) in June and 0.29 (range: 0–0.64) in September.

**FIGURE 2:**
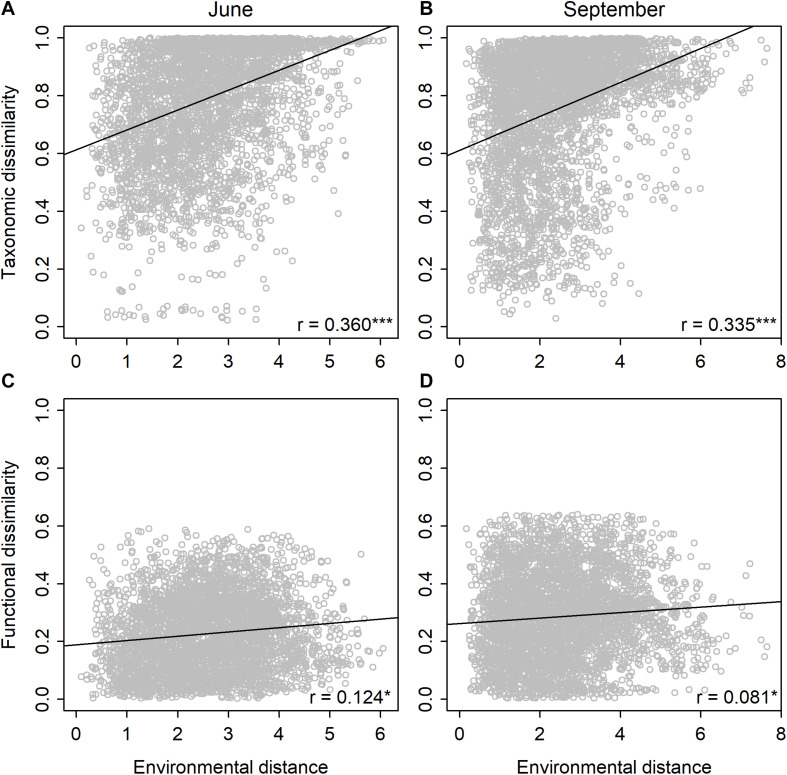
Relationships between taxonomic community dissimilarity (Bray–Curtis) and environmental distance (Euclidean) in **(A)** June and **(B)** September, and between functional community dissimilarity (Bray–Curtis) and environmental distance (Euclidean) in **(C)** June and **(D)** September for diatoms among the rock pools (*n* = 91). Shown are Mantel correlations and their statistical significances (*P < 0.05 and ***P < 0.001) and regression lines based on the linear models.

Mantel tests showed that the taxonomic community dissimilarity increased significantly (*P* < 0.001) with environmental distances in both June and September (Table 2 and Figures 2A,B). Functional community dissimilarity also increased significantly (*P* < 0.05) with environmental distance in both time periods (Table 2 and Figures 2C,D). The increase of community dissimilarity with environmental distance was consistently stronger for the taxonomic than the functional composition. Partial Mantel tests revealed that the relationships between community dissimilarity and environmental distance remained highly similar even when the effects of spatial factors were controlled for Table 2. When the effects of environmental distances were controlled for, the effects of spatial distances were significant on the taxonomic (*P* < 0.001) and functional community dissimilarities (*P* < 0.01) in September, but not in June (Table 2).

**TABLE 2:** Results of the mantel and partial mantel tests for the relationships between taxonomic and functional community dissimilarity (Bray–Curtis) and environmental distance (Euclidean) and spatial distance for diatoms in rock pools (*n* = 91).

	Environmental	Spatial	Env–Spat	Spat–Env
**Taxonomic**				
June	0.360***	0.049*	0.359***	0.040
September	0.335***	0.135***	0.327***	0.111***
**Functional**				
June	0.124*	0.047	0.123*	0.043
September	0.081*	0.107***	0.072*	0.101**

Mantel and partial Mantel correlations and their statistical significances are given (*P < 0.05, **P < 0.01, and ***P < 0.001). Env–Spat = the effects of environmental distance on community dissimilarity while controlling for spatial distance; Spat–Env = the effects of spatial distance on community dissimilarity while controlling for environmental distance.

## Discussion

To our knowledge, this is the first study to address questions about both the alpha and beta diversities of rock pool diatom communities along steep environmental gradients using a combination of taxonomic and functional approaches. We found clear patterns between the taxonomic and functional aspects of local diversity. The relationship between species richness and functional diversity was non-linear and temporally coherent such that the functional diversity initially increased more rapidly with species richness but then leveled off or increased at a decelerating rate at higher diversity levels. The results suggest that functional diversity did not increase after a certain species richness, suggesting that ∼20 species can cover most of the traits and can result in a functional redundancy above that level. However, when species richness declines below the threshold of ∼20 species, functional diversity may be rapidly lost. Hence, functional diversity and, perhaps, also ecosystem functioning may be greatly influenced by even small shifts in species diversity (Micheli and Halpern, 2005), particularly at the lower levels of taxonomic diversity.

Of the measured local explanatory variables, functional diversity was best explained by conductivity, which has also been associated with diatom functional diversity in saline (Stenger-Kovács et al., 2020) and freshwater systems (Teittinen et al., 2018). In the present study, however, variability in the functional diversity was not well captured by the measured environmental and spatial factors. Functional diversity, therefore, seems to reflect the variation in species richness more strongly than the measured explanatory variables, and the factors constraining the number of species also tend to create functionally clustered communities at low diversity levels, by selecting for species with similar traits. Indeed, the systems with a low functional diversity shared similar characteristics in that they were typically dominated by either a small, low-profile species (i.e., *Achnanthidium minutissimum*) or by small species belonging to the motile guild (e.g., *Nitzschia* spp). Ability to move allows the motile species to change their position and choose the most favorable position within the habitat (Passy, 2007), whereas *A. minutissimum* is considered a pioneer species, often forming enormous growths as the first colonizer (Cantonati et al., 2017).

In contrast to the consistent trend between species richness and functional diversity, the pattern between species richness and uniqueness of community composition was temporally inconsistent. In June, the species richness–LCBD relationship was generally negative, although slightly non-linear, and indicated that species-poor communities had a high uniqueness of species composition. To our knowledge, this is the first study to explore the local contributions to beta diversity for microorganisms among the rock pool systems. A negative association between the two metrics has also been previously observed for diatoms in other ecosystem types, such as streams (Jyrkänkallio-Mikkola et al., 2018), and for larger organisms, such as insects (Heino et al., 2017) and fish (Legendre and De Cáceres, 2013). In this study, the two metrics were best explained partly by the same variables, such that the strength of marine influence in terms of conductivity and distance to the sea seemed to create rather predictable patterns. Along a gradient of an increasing distance to the sea, species richness tended to decrease, whereas LCBD increased. LCBD also increased with decreasing conductivity. As large LCBD values denote the sites with highly disparate community compositions in the data (Legendre and De Cáceres, 2013), this outcome implies that communities with more specialist species tend to occur in the freshwater pools that are distant to the sea, whereas generalist species are relatively more common in brackish pools under a stronger marine influence. Indeed, the communities with a high uniqueness were typically dominated by freshwater species with restricted regional distributions in these data, occurring in less than one fourth of the pools (e.g., *Gomphonema parvulum*, *Nitzschia perminuta*, and *Nitzschia palea*). Freshwater pools are also relatively more isolated compared to the brackish pools located closer to the Baltic Sea, which likely presents the main permanent source of immigration to the brackish pools. Therefore, a higher exposure to colonization events induced by the occasional seawater inputs may have resulted in a higher similarity of species composition among the brackish pools. Moreover, wind dispersal rates in rock pools have been shown to be influenced by isolation, such that dispersal rates increase with decreasing distance to the nearest source populations (Vanschoenwinkel et al., 2008). The generally lower uniqueness of species compositions in brackish pools may, hence, reflect a higher connectivity to the sources of colonists and, subsequently, a higher similarity of community compositions. In addition, for passively dispersing invertebrates, more isolated rock pools have been shown to harbor less similar communities than pools linked through temporary overflows (Vanschoenwinkel et al., 2007).

In September, there was no relationship between species richness and LCBD, opposing the frequently observed negative relationship between the two aspects of diversity. Such decoupling of the two metrics points toward the differing underlying mechanisms. While the uniqueness of species composition mostly varied along the same brackish–freshwater transition as in June, the main determinants of species richness in September were the distance to the mainland and pH. The number of species decreased with an increasing distance to the mainland. This outcome may indicate the effects of the strong winds in September and the subsequently higher wind-induced wave disturbance in rock pools located on islands that were farther from the mainland. Wind exposure, which is typically higher on more distant islands (Korvenpää et al., 2003), has been shown to influence the diatom community composition in the Baltic Sea coastal region (Virta et al., 2021) and could possibly explain why species richness tends to be lower on the more distant, exposed rock pool systems. Higher levels of wave exposure have also been associated with a lower species richness of diatom communities in the northern Baltic Sea (Busse and Snoeijs, 2003). Many species-poor pools were, however, dominated by regionally widespread generalist species, which may, in part, explain the decoupling of species richness and uniqueness of community composition in September.

When we compared the level of taxonomic and functional beta diversity, we found that on average, the taxonomic beta diversity was consistently higher than the functional beta diversity, agreeing with recent observations for diatoms in the marine (Virta et al., 2020) and stream (Perez Rocha et al., 2019) systems. The increase in community dissimilarity with an increasing environmental distance was also stronger for the taxonomic than the functional composition. The increasing taxonomic dissimilarity along the environmental gradients was expected, given that the studied rock pools encompass steep environmental gradients within a relatively small spatial extent. Natural variability in environmental characteristics among the rock pools was indeed exceptionally high considering the study scale, ranging from freshwater to brackish, acid to alkaline, and oligo-mesotrophic to hypereutrophic conditions. Such high heterogeneity among the rock pools presumably allows for strong species sorting processes to operate (Leibold et al., 2004), that is, species occurring in each rock pool are filtered from the regional species pool based on the current local environmental conditions.

The results also revealed, however, that even with similar environmental conditions, taxonomic community compositions between rock pools could be highly dissimilar. Although this finding could indicate that we have missed some environmental factors that are important in structuring the diatom communities, it may also indicate that the history of community assembly affects the resulting community composition (Chase, 2003). Differences in the order and timing of species arrival may cause a divergence in the local community structure even when they have similar environmental conditions and share the same regional species pool (Fukami, 2015 and references therein). For instance, early arriving species may reduce the availability of resources to late arriving species, hence constraining their local abundances. Here, for instance, the lower species richness and functional diversity in many of the isolated freshwater pools may be indicative of such effects, especially as they were often dominated by a fast-growing pioneer species.

Compared with the taxonomic dissimilarities, the increase in functional dissimilarities with an increasing environmental distance was weaker. This outcome disagrees with a previous study in which diatom functional composition on a global scale was more strongly associated with changes in the environment compared to the taxonomic composition (Soininen et al., 2016). The outcome may, though, be dependent on the spatial extent of the study because at large spatial scales spanning distinct geographical regions, regional specificity of species composition may hinder the ability of species composition to track the environmental changes (Soininen et al., 2016). On smaller spatial extents, evidence for stronger correlations between the environment and taxonomic composition than functional composition has been found for diatoms (Virta et al., 2021), as well as other organism groups, such as macroinvertebrates (Heino and Tolonen, 2017) and fish (Villéger et al., 2012). Taken together, the results suggest that although there are apparent changes in the species composition and environmental conditions between rock pools, the functional composition of diatom communities remains rather similar. This, in turn, indicates that taxonomically disparate species present in different environments possess similar traits, resulting in relatively low functional beta diversity and weak relationships with between-site environmental heterogeneity (Heino and Tolonen, 2017). We acknowledge that the stronger taxonomic than functional distance decay may partially reflect the higher number of species compared to functional traits. We would like to note, though, that the results remained highly similar even when the taxonomic analyses where conducted using a small subset of species excluding all rare taxa and are, therefore, unlikely to be entirely contingent on the differing diversity levels between the taxonomic and functional data.

Besides the effects of environmental gradients, we found some support for the effects of spatial factors in shaping communities, agreeing with earlier studies on microorganisms in rock pool systems (Langenheder and Ragnarsson, 2007; Aarnio et al., 2019). Although the spatial effect could indicate the importance of dispersal limitation on communities, deciphering whether it truly represents dispersal effects or, for instance, the effects of unmeasured environmental factors is not straightforward (Vass et al., 2020). Given that in the present study, the pure effects of the spatial factors were rather subtle and only detected in September, we suspect that rather than indicating the potential dispersal limitation, they may at least partly reflect the effects of the unmeasured environmental factors, such as wind-induced disturbances. Due to their small size, rock pool physicochemical characteristics are closely connected to the surrounding environment and climatic conditions, and may change rapidly due to, for example, wind-induced surges of seawater, the effects of which were presumably stronger in September when wind speed was higher than in June. Moreover, if the rates of change in the environmental conditions are higher than the changes in the community compositions, communities may also bear an imprint of the past environmental conditions (Andersson et al., 2014).

In summary, this work increased our understanding of the relationships between the taxonomic and functional aspects of diatom communities and their underlying determinants among rock pools on the islands of the Baltic Sea, systems which have been rarely explored for their microbial biodiversity. Conducting the study at two time periods facilitated the identification of some generalities and inconsistencies in the patterns and key drivers of the alpha and beta diversities. Species richness and functional diversity showed a temporally coherent pattern, suggesting that especially toward lower species diversity levels, even minor decreases in the number of species may result in an impaired functional diversity and, thus, a potentially decreased ecosystem functioning. Preserving not only pools with high diversity, but also species-poor ones may, however, be vital for maintaining regional biodiversity as they may harbor taxonomically unique communities, in particular, among the isolated freshwater ecosystems. For multiple aspects of local diversity, the strength of marine influence, in terms of conductivity or the distance to the Baltic Sea emerged as the key driving factors, presumably reflecting the effects of environmental filtering and dispersal-related factors in mediating the observed patterns. At both time periods, the taxonomic beta diversity was notably higher than the functional beta diversity. The changes in the taxonomic community composition along the environmental gradients were also consistently stronger than the changes in the functional composition, implying that despite changing the species composition, communities may remain functionally similar, and therefore, robust in response to environmental changes.

## Data Availability Statement

The original contributions presented in the study are included in the article/Supplementary Material, further inquiries can be directed to the corresponding author/s.

## Author Contributions

AT and LV designed the study and collected the data. AT performed the diatom analysis and statistical analyses. AT wrote the first draft of the manuscript. LV contributed to the manuscript. Both authors approved the submitted version.

## Conflict of Interest

The authors declare that the research was conducted in the absence of any commercial or financial relationships that could be construed as a potential conflict of interest.
