# Supplementary material for: Exploring Multiple Aspects of Taxonomic and Functional Diversity in Microphytobenthic Communities: Effects of Environmental Gradients and Temporal Changes
Source: Front Microbiol. 2021 May 21;12:668993. doi: 10.3389/fmicb.2021.668993 (PMC8175668; doi:10.3389/fmicb.2021.668993)
Supplement: Supplementary file 1 [file Data_Sheet_1.docx]

Supplementary Material

**
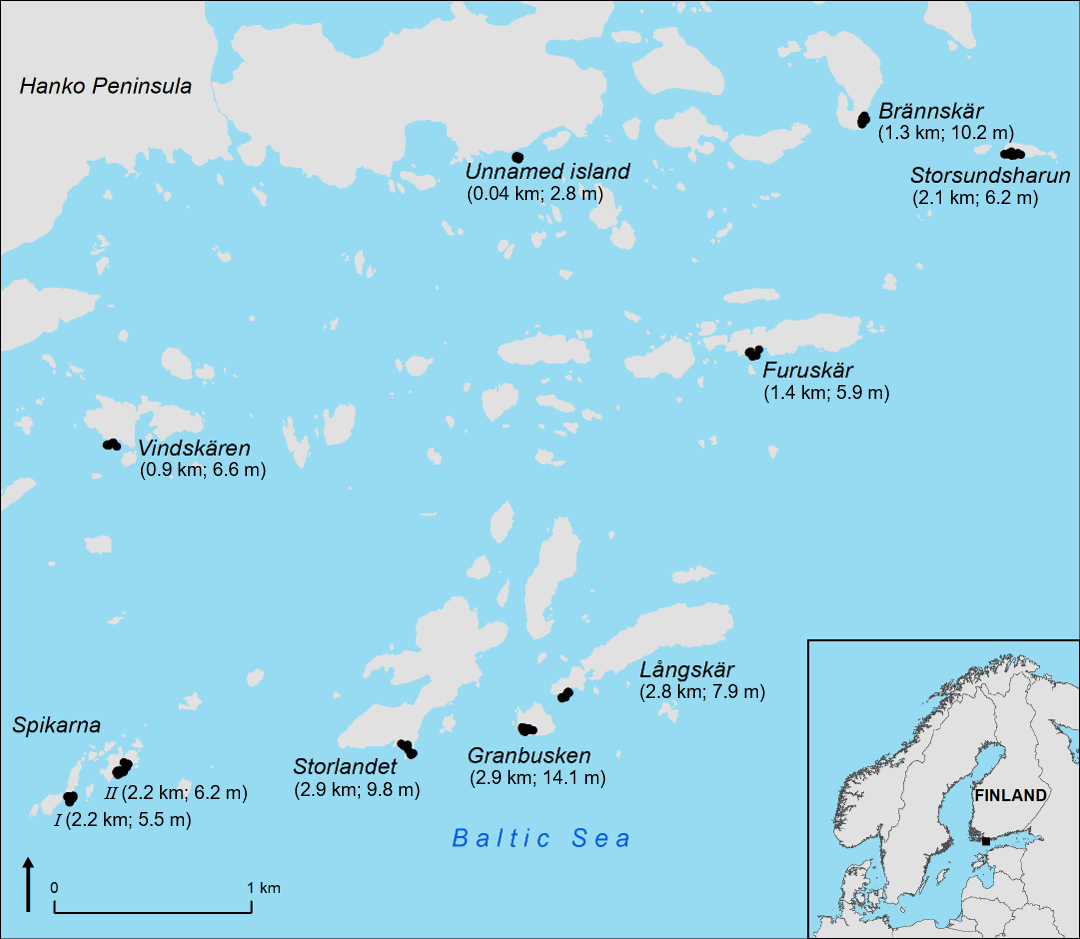
**

**Figure S1.** Locations of the 91 rock pools on ten islands of the Baltic Sea, coast of southern Finland (59°48'–59°51'N, 23°12'–23°18'E). For each island, mean distance to the mainland of Finland (km) and to the Baltic Sea (m) of the pools sampled within that island is shown. On the Spikarna island group, two islands were sampled (Ⅰ and Ⅱ). Contains data from the Finnish Environment Institute, Water formations 2016, and GADM.


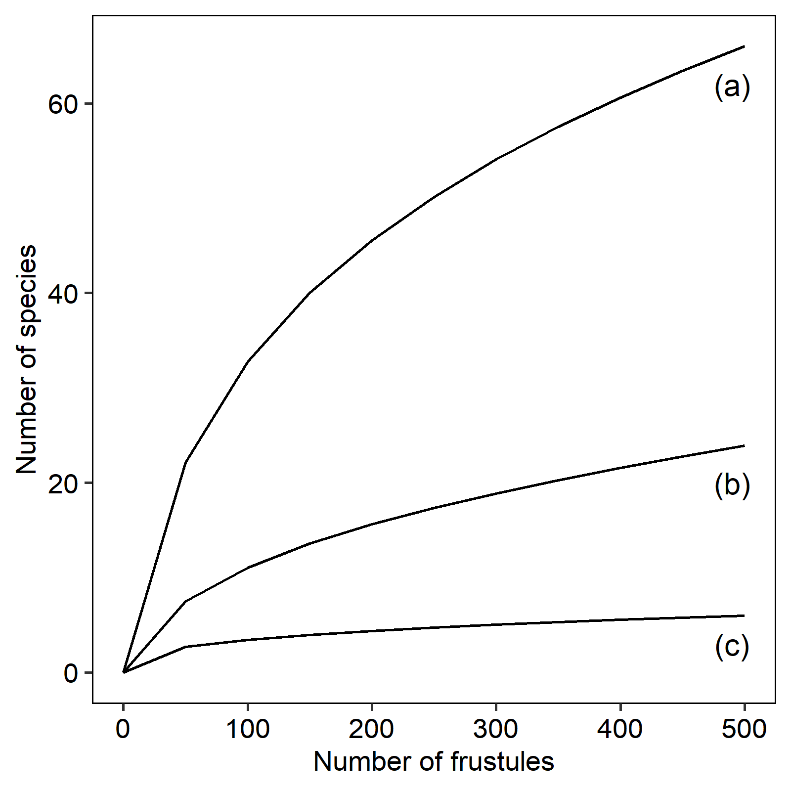


**Figure S2.** Species accumulation curves for the diatom community with (a) the highest number of species (67), (b) average number of species (24), and (c) the lowest number of species (6) in the data collected from the 91 rock pools. Each curve represents one community with either extreme or average value of species richness in these data. The community with average species richness was randomly selected from the communities that had average number of species in these data, that is, 24 species.

**
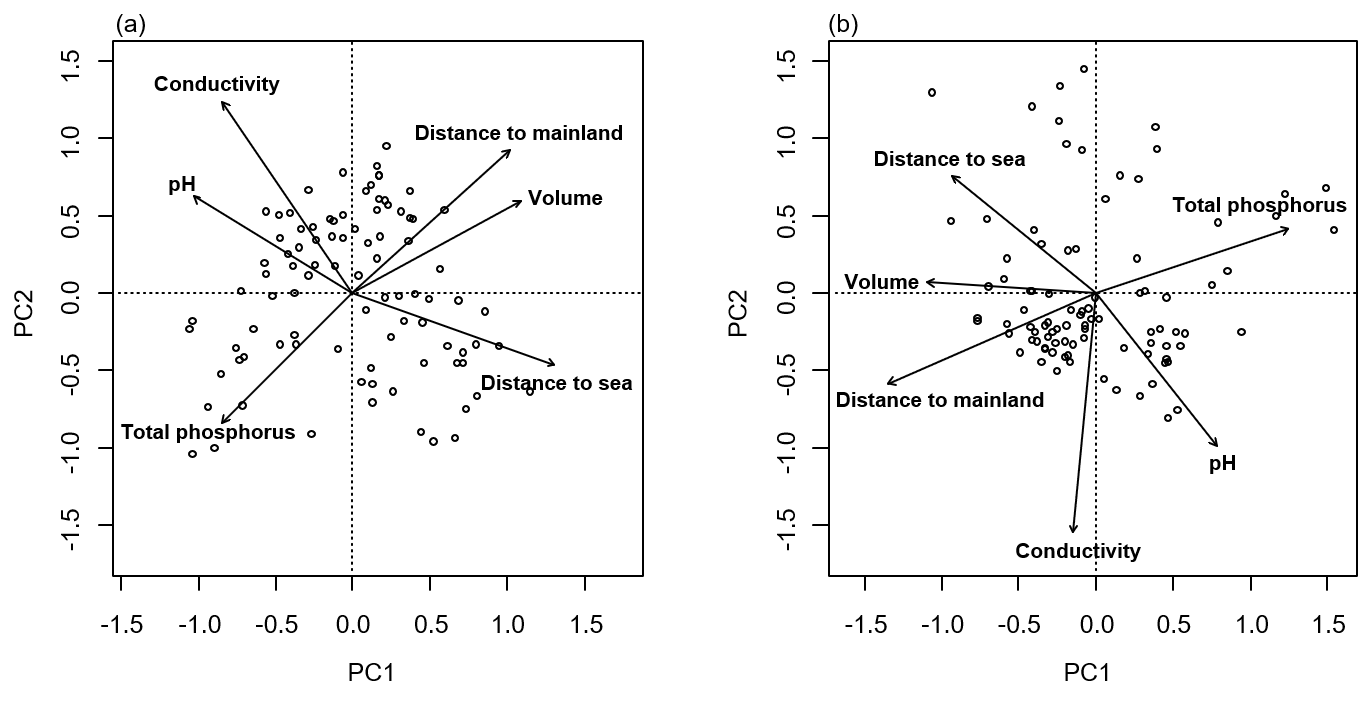
**

**Figure S3.** Principal component analysis (PCA) plots of environmental and spatial variables in (a) June and (b) September among the 91 rock pools. The first two axes explained 62.1% and 62.6% of the variation in June and September, respectively.

**Table S1.** Weather statistics for the week before sampling and during the sampling period in June and September 2018. Air temperature and wind speed represent the average of daily mean values, whereas precipitation is the sum of daily precipitation sums across the period. The air temperature and precipitation data were measured at the Tvärminne weather observation station, Hanko, and wind speed at the Tulliniemi weather observation station, Hanko (Finnish Meteorological Institute, 2020).

|  | Air temperature (°C) | Precipitation (mm) | Wind speed (m/s) |
| --- | --- | --- | --- |
| **June** |  |  |  |
| Week before sampling | 14.2 | 0.1 | 5.5 |
| During sampling | 14.5 | 26.5 | 6.7 |
|  |  |  |  |
| **September** |  |  |  |
| Week before sampling | 15.6 | 18.4 | 9.4 |
| During sampling | 11.0 | 41.0 | 9.8 |
